# *Euglossa williamsi*, a new species of orchid bee from the Amazon Basin of Ecuador and Peru, with notes on its taxonomic association and biogeography (Hymenoptera, Apidae)

**DOI:** 10.3897/zookeys.159.2239

**Published:** 2011-12-23

**Authors:** Ismael A. Hinojosa-Díaz, Michael S. Engel

**Affiliations:** 1 Division of Entomology, Natural History Museum, and Department of Ecology & Evolutionary Biology, 1501 Crestline Drive – Suite 140, University of Kansas, Lawrence, Kansas 66045, USA

**Keywords:** Apoidea, Anthophila, Euglossini, *Euglossa*, new species, taxonomy, orchid bees

## Abstract

*Euglossa williamsi* **sp. n.** is here described from the lowland Amazonian region in Ecuador and Peru, and as part of a small species assemblage within *Euglossa* consisting of *Euglossa dodsoni* Moure and *Euglossa obtusa* Dressler. An identification key to the males of the group is provided plus detailed figures of the new species and representative illustrations for the others. A brief discussion of the taxonomic and biogeographical implications of the new species is provided. New records in Honduras and Nicaragua are provided for the related *Euglossa dodsoni*.

## Introduction

With about 130 species (Nemésio and Rasmussen 2011), *Euglossa* Latreille (Apinae, Euglossini) is the most diverse of the five euglossine genera, all of them commonly known as orchid bees. The male orchid bees display a characteristic array of secondary sexual morphological features, most of them involved in the collection and handling of aromatic compounds, mainly from orchids (Dressler 1982a; Michener 2007), which are presumably used as mating attractants (Eltz et al. 2005). These same secondary sexual characters have been the basis of the taxonomic distinction among species, species groups, and the current subgeneric classification of *Euglossa* (Dressler 1978b, 1982b, c, d; Moure 1989). Despite being a taxonomically well known group of bees, as detailed revisions continue and access to newly collected material expands, particularly for infrequently visited regions,new species have continued to be discovered and described in recent years (*e.g*., Hinojosa-Díaz and Engel 2007, 2011; Hinojosa-Díaz et al. 2011; Nemésio 2007, 2011a-b; Parra et al. 2006; Ramírez 2005, 2006, Rasmussen and Skov 2006). Here we present the description of a new species from the Amazonian area of Ecuador and Peru and closely related to *Euglossa dodsoni* Moure and *Euglossa obtusa* Dressler, representing an interesting biogeographic expansion for this small cluster of species. Both *Euglossa dodsoni* and *Euglossa obtusa* are found in Central America and the western side of the Andes in Colombia. It is therefore interesting to discover a close relative on the opposing side of the Andes. An identification key to the three species is provided as well as illustrations and a discussion of their subgeneric status and the biogeographic implications of the new species.

## Material and methods

The holotype for the description of the new species belongs to the Florida Museum of Natural History, University of Florida, Gainesville, Florida, USA, while the paratype and most specimens studied of the two allied species belong to the Division of Entomology, University of Kansas Natural History Museum, Lawrence, Kansas, USA. Label data for specimens examined is provided as a detailed description of the label, with the information for each enclosed by quotation marks (“ ”), individual labels separated by double slash symbols (//), and every row on individual labels separated by a semicolon in italics (;).

Morphological terminology in general follows that of Engel (2001), Michener (2007), and Hinojosa-Díaz (2008), while some procedures for establishing metrics (*e.g*., clypeal protuberance) follow those of Brooks (1988). The species descriptions follow the overall format for other *Euglossa* species as presented by Hinojosa-Díaz and Engel (2007, 2011) and Hinojosa-Díaz et al. (2011). Photomicrographs were prepared using a Cannon EOS 7D digital camera and an Infinity K-2 long-distance microscope lens. Multilayer images were produced by using the software CombineZP.

## Systematics

### Euglossa williamsi

Hinojosa-Díaz & Engel sp. n.

urn:lsid:zoobank.org:act:0F2EC5D0-71C9-4D1C-BD57-07C80EE76AF2

http://species-id.net/wiki/Euglossa_williamsi

Figs 1 [Fig F2] 14

#### Holotype.

♂, labeled: “ECUADOR, Napo; September 1987; Dressler, Wille,; Whitten, Williams // caryophyllene; oxide". The holotype is in the Florida Museum of Natural History, University of Florida, Gainesville, Florida.

#### Paratype.

♂, labeled: “PERU: Pasco Dept,; Villa Rica-Puerto Bermudas [Bermúdez] Rd.; 910 m. 10°34'18"S, 75°5'30"W; 17 OCT 1999, D.Brzoska; D.Velasquez, PERU 1B99 047; ex: methyl salicylate // [barcode]; SM0148018; KUNHM-ENT // Euglossa; spp.; det. R. W. Brooks 19 [first two lines handwritten, year missing last two digits]”. The paratype is in the Division of Entomology, University of Kansas Natural History Museum, Lawrence, Kansas.

#### Diagnosis.

**Figures 1–2.**
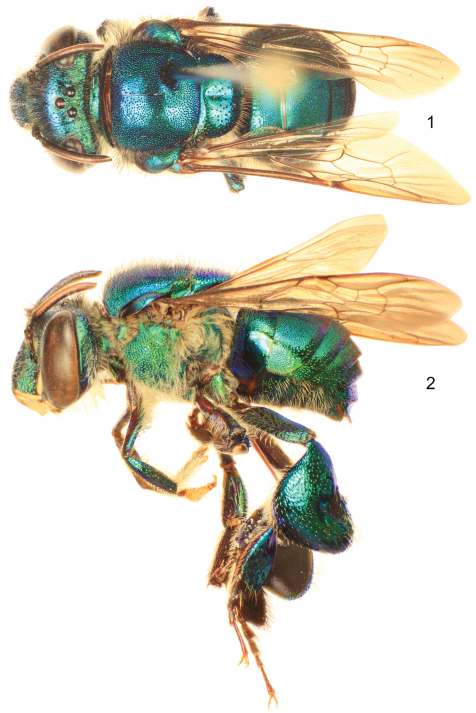
*Euglossa williamsi* Hinojosa-Díaz and Engel sp. n., male holotype **1** Dorsal habitus **2** Lateral habitus.

**Figures 3–8. F2:**
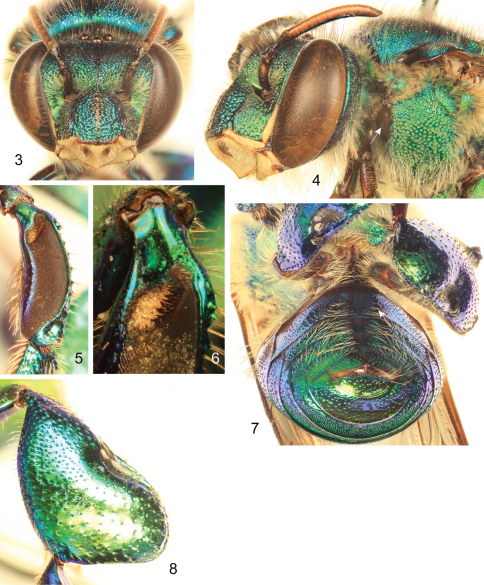
*Euglossa williamsi* Hinojosa-Díaz and Engel sp. n. male holotype **3** Facial aspect **4** Lateral aspect of mesepisterum showing *preomaular spot* (arrow) **5** Outer surface of mesotibia **6** Mesotibial tufts **7** Ventral view of metasoma showing the absence of integumental modifications on second sternum (arrow) **8** Outer view of metatibia.

Labiomaxillary complex in repose reaching sixth metasomal sternum; entire body with a dominant blue-green (teal) coloration, green iridescence, and some purple highlights (Figs 1–4, 7–8); paraocular ivory marks narrow, restricted to laterally-facing areas contiguous to compound eyes; ivory spot on antennal scape greatly reduced, present laterally on upper half of scape (Figs 3–4); anterior mesotibial tuft oblong, composed of pale, plumose setae getting darker posteriorly; posterior tuft reduced, semicircular, appearing as a continuation of anterior tuft (although differentiated from it) (Fig. 6); metatibia trapezoidal (distal third of posterodorsal margin parallel to anterior margin) (Fig. 8); second metasomal sternum with no integumental modifications (Fig. 7); punctation of mesal area of mesoscutellum, postero-ventral outer surface of metatibia, and mesal area of last two metasomal terga composed of large punctures separated by more than two puncture diameters (Figs. 1, 7–8); eighth metasomal sternum of male with posterior section triangular (lateral edges straight) (Fig. 10); dorsal process of gonocoxite broader than long; posterior margin of apical process of gonocoxite oblique (inner-posterior corner displaced posteriad) (Fig. 12); lateral area of gonostylar process of gonocoxite pronged; lateral section of gonostylus large, concave surface facing inwards and covered with dense, minutely-branched setae, gonostylar ventral lobe only differentiated apically as an acute apex (Figs. 12–14).

**Figures 9–14.**
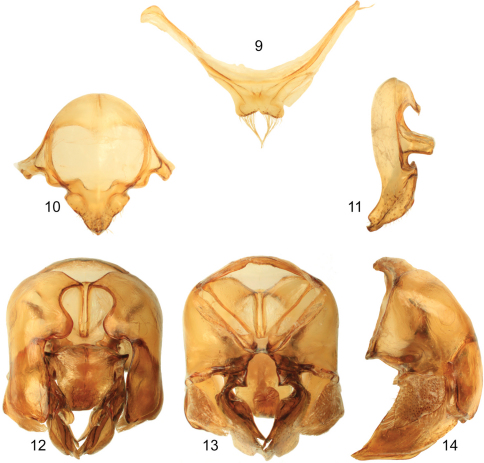
Male genitalic features of *Euglossa williamsi* Hinojosa-Díaz and Engel sp. n. **9** Seventh metasomal sternum, ventral aspect **10** Eighth metasomal sternum, ventral aspect **11** Eighth metasomal sternum, lateral aspect **12** Genitalic capsule, dorsal aspect **13** Genitalic capsule, ventral aspect **14** Genitalic capsule, lateral aspect.

#### Description.

♂: *Structure*. Total body length 10.00 mm; labiomaxillary complex in repose reaching sixth metasomal sternum. Head length 2.85 mm, width 4.30 mm; upper interorbital distance 2.00 mm; lower interorbital distance 2.00 mm; upper clypeal width 1.11 mm; lower clypeal width 1.95 mm; clypeal protuberance 0.78 mm; medial clypeal ridge well developed, paramedial clypeal ridges undistinguishable (obscured by punctation); labrum square, slightly wider than long, length 1.04 mm, width 1.11 mm; medial labral ridge sharp; paramedial labral ridges weak, oblique, almost reaching distal margin of labrum; labral windows ovoid, occupying proximal half of labrum; interocellar distance 0.30 mm; ocellocular distance 0.68 mm; first flagellomere as long (0.33 mm) as second and third flagellomeres combined (0.33 mm); length of malar area 0.15 mm. Mandible bidentate. Pronotal dorso-lateral angle slightly obliquely truncate; upper section of preomaular area with a noticeable brown, flat, polished oval surface contiguous to pronotal lobe (*preomaular spot*) (Fig. 4); intertegular distance 3.93 mm; mesoscutal length 2.48 mm; mesoscutellar length 1.26 mm; mesal area of mesoscutum concave; posterior margin of mesoscutellum weakly convex (Fig. 1); mesotibial length 2.00 mm; mesobasitarsal length 1.93 mm, width 0.67 mm (as measured at proximal posterior keel), posterior keel projected in a rounded obtuse angle; metatibial shape trapezoidal (distal third of posterodorsal margin parallel to anterior margin) (Fig. 8), metatibial anterior margin length 3.11 mm, ventral margin length 2.22 mm, postero-dorsal margin length 3.93 mm, maximum metatibial thickness 1.11 mm; metatibial organ slit narrow, basal section oval, small (length 0.37 mm), distal section spur-shaped, separated from ventral margin by its own length, maximum width occupying about one-fifth of metatibial outer surface width (Fig. 8); metabasitarsal length 1.93 mm, mid-width 0.89 mm; metabasitarsal ventral margin oblique (forming obtuse angle with anterior margin). Forewing length 8.00 mm; jugal comb with 13 blades; hind wing with 18 hamuli. Maximum metasomal width 4.07 mm; second metasomal sternum lacking integumental modifications (Fig. 7).

*Coloration*. Head with a combination of blue-green (teal) and green areas as follows: frons and clypeal disc blue-green, antennal depressions green, paraocular areas mainly green fading into blue-green along epistomal sulcus, vertex dark blue-green anteriorly, green on posterolateral sections, gena green fading into blue-green along narrow margin of compound eye and smooth lower third; hypostoma green; epistomal sulcus and medial clypeal ridge very dark, this last with faint coppery hue; paraocular ivory marks narrow, restricted to laterally-facing areas contiguous to compound eyes, ivory color surrounded by thin brown margin; lower lateral parts of clypeus ivory, amber-translucent at edge; labrum ivory; labral anterior and posterior edges as well as labral windows amber-translucent; malar area mainly ivory (brown on narrow areas of anterior and posterior extremes); mandible ivory on outer surface, teeth and ridges brown; antennal scape, pedicel and first fagellomere dark brown, remaining flagellomeres light brown on anterior surface, dark brown on posterior surface; scape with greatly reduced ivory spot, present laterally on upper half (Figs 3–4). Pronotum green/blue-green iridescent, appearing mainly blue-green on anterior section of dorsum and dark blue-green on anterior-facing surface of pronotal lobe; mesoscutum, mesoscutellum, and tegula blue-green with green iridescence (Figs 1–2); lateral-facing surface of mesepisternum mainly green with blue-green iridescence (mainly blue-green on upper area contiguous to pronotal lobe) (Fig. 4); preomaular area with a large brown oval-shaped area (preomaular spot) on upper half (Fig. 4), otherwise blue-green; metepisternum and propodeum green with blue-green iridescence; pro- and mesocoxa and pro- and mesotrochanter brown with strong blue-green iridescence; profemur, protibia and probasitarsus with dominant blue-green iridescence; mesofemur with some purple iridescence on anterior surface otherwise with blue-green, mesotibia similarly colored except purple coloration stronger, mesobasitarsus blue-green on outer surface; hind leg with all podites (except metatarsomeres beyond metabasitarsus) blue-green on outer surfaces and few purple highlights; tarsomeres beyond basitarsi of all legs brown, pretarsal claws with yellow shaft and brown tips (Fig. 2); wings hyaline with brown veins and light green and coppery hue (Figs 1–2). First to fourt hmetasomal terga blue-green with green iridescence; fifth to seventh terga mainly green, all with faint purple highlights (highlights stronger on ventrolateral sections of first metasomal tergum) (Figs 1–2); sterna green with blue-green iridescence mesally and golden iridescence laterally (Fig. 7).

*Sculpturing*. Face densely areolate-punctate, areole-punctures strong, about one-third median ocellar diameter on clypeal disc, smaller on frons (nearly one-tenth of median ocellar diameter) (Fig. 3); vertex on anterior ocellar area smooth with scattered round punctures; gena shallowly areolate, smooth on a narrow streak close to compound eye (except for scattered large punctures on upper margin) and particularly on lower area close to hypostoma. Mesoscutum and mesoscutellum punctate, mesoscutal puncture size about one-quarter of median ocellar diameter on anterolateral corners and posterior margin, where they are also denser (contiguous), punctures smaller (about one-eighth of median ocellar diameter) and sparser (separated by at least one puncture diameter) on mesal area along median mesoscutal line, intermixed with some very minute punctures (Fig. 1); mesoscutellar punctures of two sizes, most as big as about one-third of median ocellar diameter, intermixed with scarcer minute punctures, punctation denser along posterior margin (contiguous punctures), and sparse on mesal area of mesoscutellar disc where punctures are separated by more than two puncture diameters leaving large smooth areas (Fig. 1); mesepisternal lateral-facing surface with sculpturing similar to that on lower frons or clypeal disc, punctures becoming slightly larger towards venter (Figs 2, 4); preomaular area with shallow punctures on metallic integument (not on preomaular spot), preomaular spot with polished edge and smooth-minutely imbricate main central area (Fig. 4); metatibial punctures equivalent in size to those on mesoscutellar central area, denser (separated by no more than a puncture diameter) along anterior margin, getting sparser (separated by two to three puncture diameters) towards posterior area, such that there is smooth integument on contact area with metatibial organ slit (Fig. 8). First metasomal tergum with anterolateral corners sculpturing comparable to that on anterolatreral corners of mesoscutum, quarter along anterior margin punctures as on mesoscutellar disc, posterior three-quarters densely punctate, punctures shallow, slightly smaller than those on median area of mesoscutum, leaving a narrow smooth area along posterior margin, ventrolateral sections polished; second to fourth terga with punctation as on posterior three-quarters of first tergum except larger and sparser punctures on lateral bending areas; anterior portion of fifth tergum with punctation as on preceding terga, punctures becoming progressively larger posteriorly, mesal section with median longitudinal smooth area; sixth and seventh terga punctures considerably larger and sparser, punctation comparable to that on mesoscutellum (Fig. 7); first metasomal sternum smooth; second metasomal sternum with anterolateral and lateral areas next to margins of contacting terga smooth, otherwise punctation comparable to that on sixth tergum, except mesally where punctures become smaller and shallower, posterior margin with smooth band all along; remaining sterna similarly punctuate, except denser punctures (Fig. 7).

*Vestiture*. Frontal fringe composed of two kinds of dense setae, some dark brown, minutely serrate, others fulvous, plumose, both evenly combined and about as long as two mid-ocellar diameters; clypeus and labrum with scattered, shorter brown and light setae, appearing simple, mandibular outer surface with similar setae but shorter; paraocular areas with moderately dense, pale, minutely-branched setae, becoming longer towards epistomal sulcus, area contiguous to upper section of epistomal sulcus with moderately dense, dark brown, minutely-serrate setae, as long as fringe setae; antennal depressions with dense, appressed, fulvous, plumose setae; vertex with scattered, fulvous, simple, minute setae on smooth area anterior to ocelli, some scattered, dark, simple setae on lateral areas contiguous to compound eye margin, central area of ocellar triangle and posterior section of vertex with moderately dense, dark, minutely-serrate, long setae, those on posterior margin nearly twice as long as those on fringe and intermixed with some shorter, fulvous, simple setae; gena with dense, fulvous, plumose setae, appearing simple on upper posterior area and increasing in size towards lower genal section, continuous with simpler setae along ventral mandibular margin, some scattered, dark, simple, short setae along compound eye margin; antennal scape and pedicel with scattered, dark, short, simple setae; flagellum covered with dense, fulvous, simple, minute setae (Figs 3–4). Mesoscutum and mesoscutellum covered with combination of setae similar to that of frontal fringe, slightly sparser and distributed in same pattern described for punctation; pronotal lobes densely covered by fulvous, plumose setae as long as those on fringe, intermixed with dark, minutely-serrate setae; lateral-facing surface of mesepisternum, metepisternum and propodeum covered by, dense, fulvous, plumose setae as long as those on frontal fringe, preomaular area uncovered on preomaular spot, otherwise with dense, fulvous, plumose setae shorter than those on lateral areas of mesosoma (Fig. 4); foreleg with moderately dense fulvous setae from procoxa to protibia, short and appearing simple on most surfaces, except posterior surfaces of profemur (especially) and protibia, where setae are of same nature as on lateral areas of mesosoma; basitarsus with dense, yellowish, sturdier setae on inner surface; chemical gathering tufts on second through fourth protarsomeres made of dense, orange, long, setae; mid- and hind legs with general vestiture similar to foreleg except as follows: coxae with dense plumose setae; basitarsi with dense, brownish, sturdy clothing on inner surfaces (mesobasitarsus with three major wavy setae); mesotibia with dense, simple, yellowish setae directed downwards on anterior surface, shorter scattered setae on posterior surface, microtrichia on outer mesotibial surface (velvety area) composed of dense, fulvous, simple, minute setae; anterior margin of velvety area concave, anterior mesotibial tuft oblong, diagonally oriented, composed of dense, pale, plumose setae becoming darker posteriorly; posterior tuft reduced, appearing as a semicircular posterior continuation of anterior tuft (although differentiated from it) (Fig. 6); metatibia with rather scattered setae on outer surface; metatibial organ slit closed with brown setae (Fig. 8). Metasomal terga appearing bare, but covered with dense, fulvous, simple minute setae with some scattered, dark sturdier setae, anterolateral corners of first tergum, lateral sections of second and third terga, and posterior margin of seventh tergum with long setae; second to sixth metasomal sterna covered with moderately dense, fulvous, simple, long setae, as long as those on frontal fringe, becoming sparser towards mesal area (Fig. 7).

*Terminalia*. Seventh metasomal sternum with posterior invagination bearing a fringe of simple setae on each side of invaginated area (Fig. 9); eighth metasomal sternum with posterior section triangular (dorsal or ventral view), lateral edges of posterior section straight or at most very shallowly invaginated (Fig. 10); posterior section covered with scattered, short, simple setae; dorsal surface of posterior section strongly convex (Fig. 11). Dorsal process of gonocoxite broader than long, appearing more like a semicircle; posterior margin of apical process of gonocoxite oblique (inner-posterior corner displaced posteriad) (Fig. 12); lateral area of gonostylar process of gonocoxite pronged; spatha surface with transversal-diagonal striae; lateral section of gonostylus large, extended with a concave surface facing inwards and covered with dense, minutely-branched setae, gonostylar ventral lobe not very well differentiated from whole lateral section, but extended apically as an acute apex (Figs 12–14).

♀: Unknown.

#### Etymology.

The specific epithet is a patronym honoring Dr. Norris Williams, curator at the Florida Museum of Natural History, who granted access to the holotype and was also part of the team that collected it, and in recognition of his numerous contributions toward understanding the euglossine fauna.

### Euglossa dodsoni

Moure

http://species-id.net/wiki/Euglossa_dodsoni

#### New records.

1♂, labeled: “NICARAGUA: Rio San Juan Dept.; 60 km SE San Carlos, Refugio; Bartola, 100m, 10°58.40'N, 84°20.30'W; 28-V-2002, R. Brooks, Z. Falin,; S. Chatzimanolis ex. methyl salicylate/; eucalyptus oil baits, NIC1BFC02111 // [barcode]; SM0534510; KUNHM-ENT // Euglossa; dodsoni; Moure; Det. I. Hinojosa-Díaz 2004 [specific epithet and author handwritten]". 1♂, labeled, “HONDURAS: G.a Dios; Krausirpi; 15°03'N, 84°52'W; 21–24.V.1994; B.D. Gill // Euglossa; dodsoni ♂; Moure; det. R.W. Brooks 1995 [all handwritten except determiner and first two digits of year]. 2♂♂, locality label as in preceding specimen, determination label: “Euglossa; dodsoni; Moure; Det. I. Hinojosa-Díaz 2011 [specific epithet and author handwritten]". All deposited in the Division of Entomology, University of Kansas Natural History Museum, Lawrence, Kansas.

#### Key to Species Allied to *Euglossa dodsoni* (males only)

**Table:** 

1	Ivory spot on antennal scape greatly reduced, present laterally on upper half of scape (Figs 3–4); mesoscutellum with sparse punctures on mesal area (punctures separated by at least three puncture diameters), leaving large smooth integumental portions among punctures (Fig. 1); entire body with dominant blue-green (teal) coloration (Figs 1–2) (Amazonian Ecuador & Peru)	*Euglossa williamsi* sp. n.
–	Ivory spot on antennal scape well developed, occupying most of antero-lateral surface of scape (Figs 16, 19); mesoscutellum with moderately dense to dense punctures on mesal area (punctures separated by no more than two puncture diameters) (Figs 15, 18); body coloration either green with strong coppery-reddish iridescence or mainly green	2
2	Body coloration green but with a strong coppery-reddish iridescence, particularly on mesoscutum, mesoscutellum, and anterior two-thirds of metasomal terga (Fig. 15) (Honduras to Pacific lowlands of Ecuador)	*Euglossa dodsoni* Moure
–	Body coloration green, at most with some faint coppery iridescence mainly on mesoscutum and anterior half of second metasomal tergum (Fig. 18) (Southern Mexico and Belize)	*Euglossa obtusa* Dressler

**Figures 15–17.**
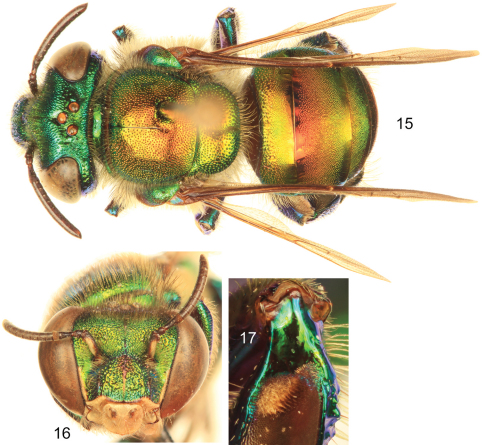
*Euglossa dodsoni* Moure, male. **15** Dorsal habitus **16** Facial aspect **17** Mesotibial tufts.

**Figures 18–20.**
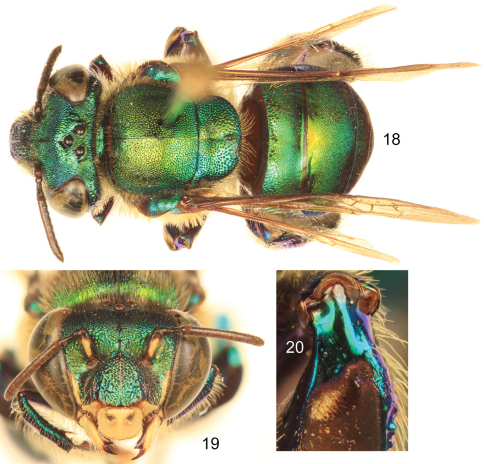
*Euglossa obtusa* Dressler, male.**18** Dorsal habitus **19** Facial aspect **20** Mesotibial tufts

## Discussion

*Euglossa williamsi* is closely related to both *Euglossa dodsoni* and *Euglossa obtusa* as evidenced by the basically identical morphology of the mesotibial tufts, metatibial shape, and genitalic structures in all three species, as well as by the absence of integumental modifications on the second metasomal sternum and the noticeable preomaular spot (integumental area on the preomaular area of the mesepisternum), features that can be considered as diagnostic for the small species assemblage. Moure (1965) originally described *Euglossa dodsoni* as a species in *Euglossa* *s. str*. and alluded to a close relationship of this species with *Euglossa purpurea* Friese. Moure’s assertion must be seen in the context of the subgeneric classification of *Euglossa* *s. lat*. at the time, although the association of *Euglossa dodsoni* to *Euglossa purpurea* was most likely based exclusively on coloration given that both species belong to rather distinct and separate lineages within the genus. Dressler (1978a) described *Euglossa obtusa* as part of the subgenus *Glossura* Cockerell, again in agreeance with the subgeneric classification of the genus at that time. Dressler (1978a) also noted that *Euglossa obtusa* was closely related to *Euglossa dodsoni*, from which he distinguished it mainly on coloration and sculpturing while leaving open the possibility that they were conspecific. The subgeneric placement of *Euglossa dodsoni* and *Euglossa obtusa* was redefined to its present usage by Dressler (1982c) and as part of the subgenus *Glossurella* Dressler, which was established to encompass a complex and diverse species group [*i.e*., those species allied to *Euglossa bursigera* Moure and originally recognized as an informal group in Dressler’s original infrageneric classification (Dressler 1978b)]. In accordance with the currently employed system of subgenera, assignment of *Euglossa williamsi* would be to *Glossurella*. However, recent phylogenetic treatments of the genus based both on morphology (Hinojosa-Díaz 2010, in prep.) and on DNA sequence (Ramírez et al. 2010) evidence recovers *Glossurella* as paraphyletic. Despite the non-monophyletic nature of *Glossurella*, *Euglossa dodsoni* and *Euglossa obtusa* are recovered as sisters in both phylogenetic analyses. Because *Glossurella*, as it is currently understood, is not a natural group in either analysis its usage under a phylogenetically congruent classification should be avoided, and as such we prefer to view *Euglossa williamsi*, as well as *Euglossa dodsoni* and *Euglossa obtusa*, as *incertae sedis* in regard to subgeneric assignment within *Euglossa*.

*Euglossa dodsoni* and *Euglossa obtusa* are very similar, including in their male terminalia, only differentiated on the basis of body coloration and puncture density (although this last feature is somewhat dubious and not so evident) as asserted by Dressler (1978a). Given the few features distinguishing the two Dressler (1978a) cautioned that it was possible that they were conspecific. We prefer to treat them here as separate species based on the stable expression of the body coloration pattern across the distributional range of both taxa.

The presence of the rather large preomaular spot constitutes an additional morphological featured shared by the three species (*Euglossa dodsoni*, *Euglossa obtusa*, and *Euglossa williamsi*) that can be used to easily characterize their males. Interestingly, despite the large and conspicuous nature of this trait, it was not mentioned in the original or any subsequent descriptions of *Euglossa dodsoni* or *Euglossa obtusa* (Dressler 1978a; Moure 1965). This distinctive preomaular area occurs widely in euglossines but is typically very small, only being expanded so within this small species group.

The two previously known species of this small group are distributed from southern Mexico to Colombia. The northernmost species, *Euglossa obtusa*, is known from a few localities in the lowlands of southeastern Mexico and Belize, both from literature records (Dressler 1978a) and specimens reviewed by the authors, while *Euglossa dodsoni* was described originally by Moure (1965) from Costa Rica and Panama, but is mentioned to occur also in Colombia (Bonilla-Gómez and Nates-Parra 1992; Ramírez et al. 2002; Roubik and Hanson 2004) and Ecuador (Ramírez et al. 2002). For this last species we reviewed specimens from Costa Rica, Panama, Nicaragua, and Honduras, extending the distributional records of this species northwards (based on these last two countries, *vide supra*). The distribution of *Euglossa dodsoni* in northwestern South America is here inferred to follow the lowlands along the Pacific Coast, west of the Andes. Ramírez et al. (2002) mention this species to be found in the Colombian Pacific region, in accordance with the aforementioned distributional range, and, although we cannot be completely certain, the Ecuadorian records mentioned by these authors most likely follow the same pattern (*i.e*.,present on the Pacific Coast, absent on the Amazonian side of the country). Some other species groups within *Euglossa* *s. lat*. have a similar distributional pattern (as do some Meliponini), specifically the group of species allied to *Euglossa (Euglossella) cyanura* Cockerell (Hinojosa-Díaz and Engel in prep.). The locality data for the holotype of *Euglossa williamsi* does not give enough information beyond the province in Ecuador where it was collected, Napo, which at the time of the collecting event included the current provinces of Sucumbios and Orellana. A survey of the entire database of orchids collected at about the same time and from the same place present in the Florida Museum of Natural History revealed similar label data and no further insights into a more precise location. Despite the challenge of assigning the specimen to a more specific locality, it is certain that it was collected on the eastern side of the Andes since the Provinces of Napo, Sucumbios, and Orellana are all on the Amazonian side of Ecuador (east of the Andes). Given that the paratype was captured in the Amazonian lowlands of Peru suggests the same was likely the case for the Ecuadorian holotype. This is an interesting addition to the overall distribution of this small species group as it represents the presence of one taxon within a different larger biogeographic unit.

## Supplementary Material

XML Treatment for Euglossa williamsi

XML Treatment for Euglossa dodsoni
